# Single-cell transcriptomic analysis of vascular endothelial cells in zebrafish embryos

**DOI:** 10.1038/s41598-022-17127-w

**Published:** 2022-07-29

**Authors:** Suman Gurung, Nicole K. Restrepo, Brendan Chestnut, Laurita Klimkaite, Saulius Sumanas

**Affiliations:** 1 Division of Developmental Biology, Cincinnati Children’s Hospital Medical Center, Cincinnati, OH USA; 2 Department of Pediatrics, University of Cincinnati College of Medicine, Cincinnati, OH USA; 3 Department of Pathology and Cell Biology, USF Health Heart Institute, University of South Florida, 560 Channelside Dr, Tampa, FL 33602 USA

**Keywords:** Angiogenesis, Developmental biology, Zebrafish

## Abstract

Vascular endothelial cells exhibit substantial phenotypic and transcriptional heterogeneity which is established during early embryogenesis. However, the molecular mechanisms involved in establishing endothelial cell diversity are still not well understood. Zebrafish has emerged as an advantageous model to study vascular development. Despite its importance, the single-cell transcriptomic profile of vascular endothelial cells during zebrafish development is still missing. To address this, we applied single-cell RNA-sequencing (scRNA-seq) of vascular endothelial cells isolated from zebrafish embryos at the 24 hpf stage. Six distinct clusters or subclusters related to vascular endothelial cells were identified which include arterial, two venous, cranial, endocardial and endothelial progenitor cell subtypes. Furthermore, we validated our findings by characterizing novel markers for arterial, venous, and endocardial cells. We experimentally confirmed the presence of two transcriptionally different venous cell subtypes, demonstrating heterogeneity among venous endothelial cells at this early developmental stage. This dataset will be a valuable resource for future functional characterization of vascular endothelial cells and interrogation of molecular mechanisms involved in the establishment of their heterogeneity and cell-fate decisions.

## Introduction

Vascular endothelial cells in different vascular beds exhibit considerable heterogeneity. This endothelial cell diversity is established during early embryogenesis when vascular endothelial cells are specified from mesodermal progenitors and differentiate into arterial, venous, capillary, lymphatic, endocardial, hemogenic and other subtypes of endothelial cells^1^. Despite advances in our understanding of mechanisms that govern vascular development, the extent of vascular heterogeneity and molecular mechanisms involved in its establishment are still poorly understood.

Because it is technically challenging to study embryonic vascular development in mammalian embryos, the zebrafish has emerged as an advantageous model to study early vascular development. Transparent zebrafish embryos develop externally and allow researchers to study spatiotemporal events in vivo and in real time. Furthermore, the signaling pathways and transcriptional programs that regulate early vascular development are highly conserved between zebrafish and other vertebrates^2^.

The advent of large-scale single cell RNA-sequencing (scRNA-Seq) in recent years has enabled robust access to the transcriptomic profile of single cells. Detailed analysis of scRNA-Seq data has revealed similarities and differences between different cell types and allowed researchers to carry out analysis of complexity and heterogeneity existing in the biological systems^3,4^. Numerous studies involving single-cell studies in health and disease have identified new endothelial cell subtypes and functions^5–10^. Several scRNA-Seq studies using zebrafish embryos have revealed cellular heterogeneity and developmental trajectory during tissue and organ development in zebrafish^11^. In addition, a single cell transcriptomic atlas of day one to five zebrafish embryos has been generated recently^12^. However, the transcriptional profile of zebrafish vascular endothelial cells, which might provide insights into vascular endothelial cell heterogeneity and cell fate decision process during zebrafish development, is still missing.

Kinase insert domain receptor like *(Kdrl),* also termed as *flk1*, is strongly expressed in developing endothelial precursors and differentiated vascular endothelial cells in zebrafish embryos^13^. We have previously generated *Tg(kdrl:mCherry)* transgenic line which faithfully recapitulates endothelial *kdrl* expression in zebrafish embryos^14^. Etv2/Etsrp, an ETS transcription factor, is one of the earliest markers of vascular progenitor cells in multiple vertebrates including zebrafish^15–17^. We have previously generated *etv2*^*ci32Gt*^ gene trap line which has an insertion of the Gal4 transcriptional activator within the *etv2* genomic locus. Furthermore, *etv2*^*ci32Gt*+*/−*^*; UAS:GFP* embryos are phenotypically normal and faithfully recapitulate GFP expression in vascular endothelial, myeloid and red blood cells^18^.

Here, we performed scRNA-seq using a combination of *Tg(kdrl:mCherry)* and *etv2*^*ci32Gt*^*;UAS:GFP* reporter lines to generate transcriptomic profile of vascular endothelial cells isolated from zebrafish embryos at 24 hpf (hours post fertilization) stage, when blood circulation is first initiated. We identified transcriptional profile of 6 different subtypes of vascular endothelial cells, which include vascular progenitor, arterial, two different venous, endocardial, and mixed arteriovenous subtypes. We validated our findings by characterizing novel markers for arterial, venous, and endocardial cells. We experimentally confirmed the presence of two transcriptionally different venous cell subtypes, demonstrating unexpected heterogeneity among venous endothelial cells at this early developmental stage. This data provides a valuable resource for future functional characterization of vascular endothelial cells and interrogation of molecular mechanisms involved in the establishment of their heterogeneity.

## Results

**Figure 1:**
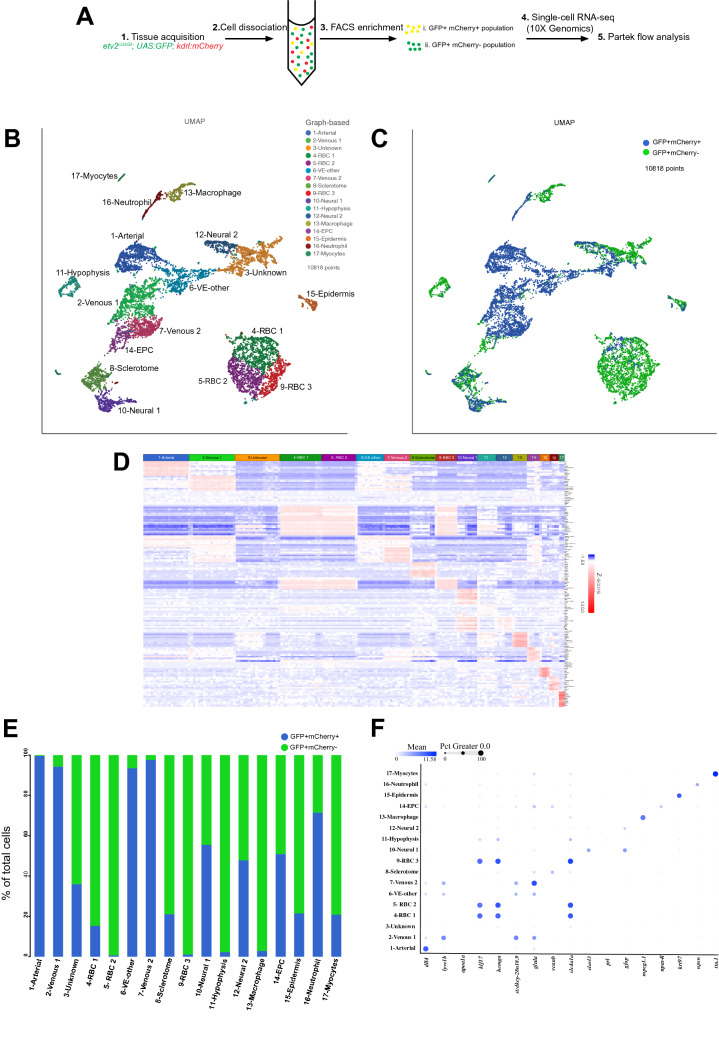
The scRNA-seq analysis of *etv2*^*ci32Gt*^*; UAS:GFP*; *kdrl:mCherry* embryos at the 24 hpf stage. (**A**) Schematics of the experimental design for scRNA-seq. (**B**) UMAP plot of 10,818 cells with 17 distinct clusters. Classifications were based on previously known marker genes which were significantly enriched in each cluster. RBC, red blood cells; VE, vascular endothelial; EPC, endothelial progenitor cells. (**C**) UMAP plot showing the distribution of GFP + mCherry + and GFP + mCherry- cells. (**D**) A heatmap showing expression of top marker genes in different clusters. Enlarged heatmap and gene list is shown in Fig. S1. (**E**) Fraction of GFP + mCherry + and GFP + mCherry- cells within each cluster. (**F**) A dot plot showing the expression of selected marker genes in different clusters.

To obtain a single-cell transcriptional profile of vascular endothelial cells, we crossed the previously established *Tg(kdrl:mCherry)* and *etv2*^*ci32Gt*^*; UAS:GFP* lines^14,18^. GFP and mCherry positive embryos were collected at 24 hpf. Because the *etv2*^*ci32Gt*^*; UAS:GFP* line labels vascular endothelial and some other cell types, such as blood, we decided to collect two different cell populations to help with subsequent annotation and line characterization. Thus, GFP + mCherry + and GFP + mCherry- cell populations were isolated by FACS (fluorescent activated cell sorting) and subjected to single-cell RNA-seq analysis using the Chromium Controller (10 × Genomics) followed by the next-generation sequencing (Fig. 1A). After filtering, transcriptomes of 5275 and 5543 cells were obtained from GFP + mCherry + and GFP + mCherry- cell populations, respectively. Transcriptomes from both cell populations were pooled for further analysis. Unbiased graph-based clustering was performed using the Partek flow analysis package, and 17 distinct clusters were identified and visualized using Uniform Manifold Approximation and Projection (UMAP) approach (Fig. 1B,C). We then assigned cell identities to these clusters based on previously known marker genes which were significantly enriched in each cluster and showed specific gene expression patterns (Fig. 1B–F, Fig. S1 and Tables S1, S2). This revealed major cellular subtypes including five vascular endothelial, three red blood cell (RBC), sclerotome, two neural, macrophage, endothelial progenitor cell (EPC), epidermis, neutrophil, and myocytes. Vascular endothelial cells were mostly GFP + mCherry +, while RBC and macrophage clusters were largely GFP + mCherry− (Fig. 1E). This is consistent with known *etv2* reporter expression in both endothelial and blood cells^18^, while *kdrl:mCherry* expression is largely restricted to vascular endothelial cells^14^.

Five distinct clusters related to vascular endothelial cells including vascular progenitor, arterial, two different venous, and mixed arteriovenous subtypes were obtained, underscoring the heterogeneity of vascular endothelial cells. We discuss these clusters in greater detail below.

### Identification of an arterial cluster

**Figure 2:**
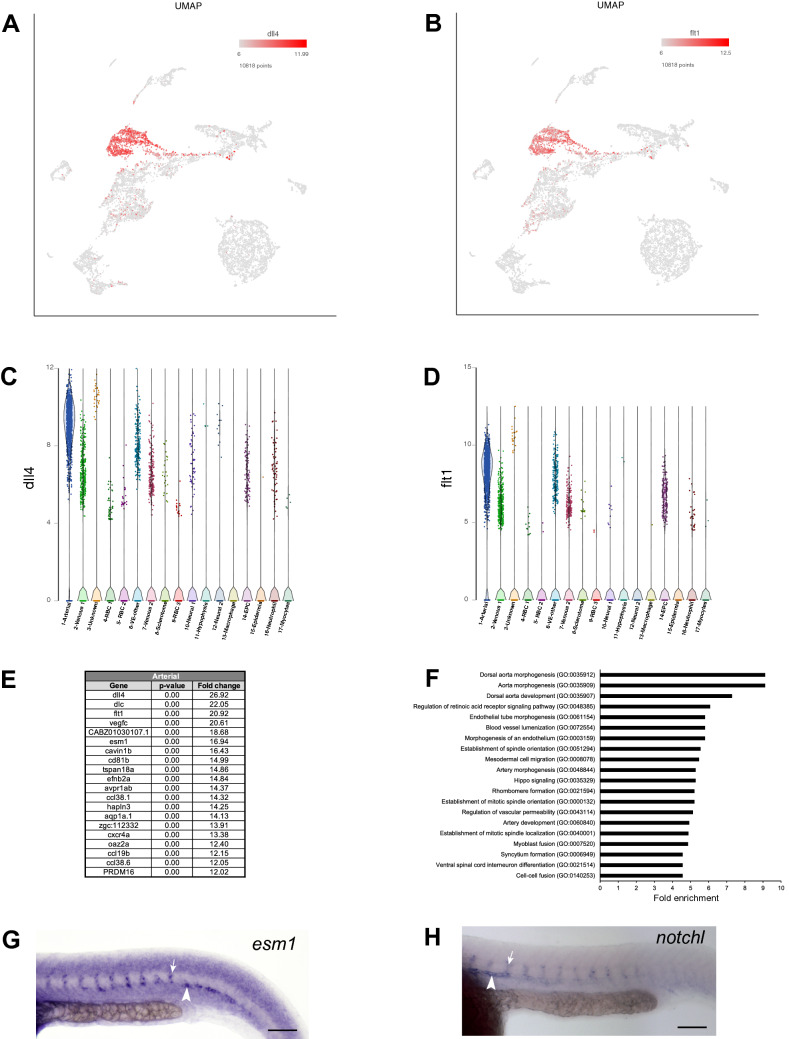
Arterial cluster #1. (**A**,**B**) UMAP feature plots showing expression of selected arterial top markers *dll4* and *flt1*. (**C**,**D**) Violin plots showing expression of *dll4* and *flt1* in different cell populations. (**E**) List of top 20 marker genes differentially expressed in arterial cluster. (**F**) List of top 20 pathways enriched in arterial cluster. (**G**,**H**) In situ hybridization analysis at 24 hpf for selected marker genes *esm1* and *notchl*. Note the expression of *esm1* and *notchl* in the dorsal aorta (arrowhead) as well as the intersegmental vessels (arrow). Scale bars: 100 μm.

Top marker genes for cluster #1 included *dll4*, *dlc*, and *flt1*, which are known to be expressed in the arterial cells^19–21^ (Fig. 2A–E and Table S1). Many other known arterial markers, including *vegfc, aqp1a.1* and *hey2,* were also enriched in cluster #1^20,22,23^ (Fig. 2E and Table S1). In addition, cluster #1 also showed increased expression of unknown or previously uncharacterized genes. We hypothesized that many of these genes would be also expressed in arterial cells. Indeed, in situ hybridization (ISH) analysis of two selected previously uncharacterized genes, *esm1* and *notchl,* demonstrated their expression in the dorsal aorta and intersegmental vessels (ISV) (Fig. 2G,H and Table S1). *esm1* expression appeared enriched in the sprouting ISVs and was reduced in non-sprouting segments of the DA, suggesting further heterogeneity within the arterial population. In an attempt to gain insights into the biological processes represented by enriched genes in cluster #1, we performed Gene ontology (GO) and Pathway enrichment analysis. Top GO biological processes represented by gene set in cluster #1 included dorsal aorta morphogenesis, aorta morphogenesis, and dorsal aorta development (Fig. 2F and Table S3). Furthermore, pathway analysis revealed enrichment of Hedgehog signaling pathway, known to play an important role in arterial development^24^, including the regulation of size of the dorsal aorta^25^ (Table S4). Other enriched pathways included histamine H1 receptor signaling pathway, which is known to regulate arterial vasodilation^26^, as well as PI3 kinase, integrin signaling and EGF receptor signaling pathways (Table S4), which have been implicated in several steps of vascular development including angiogenesis^27^, vasculogenesis^28^ and arterial development^29,30^.

### Identification of two subtypes of venous cells

**Figure 3:**
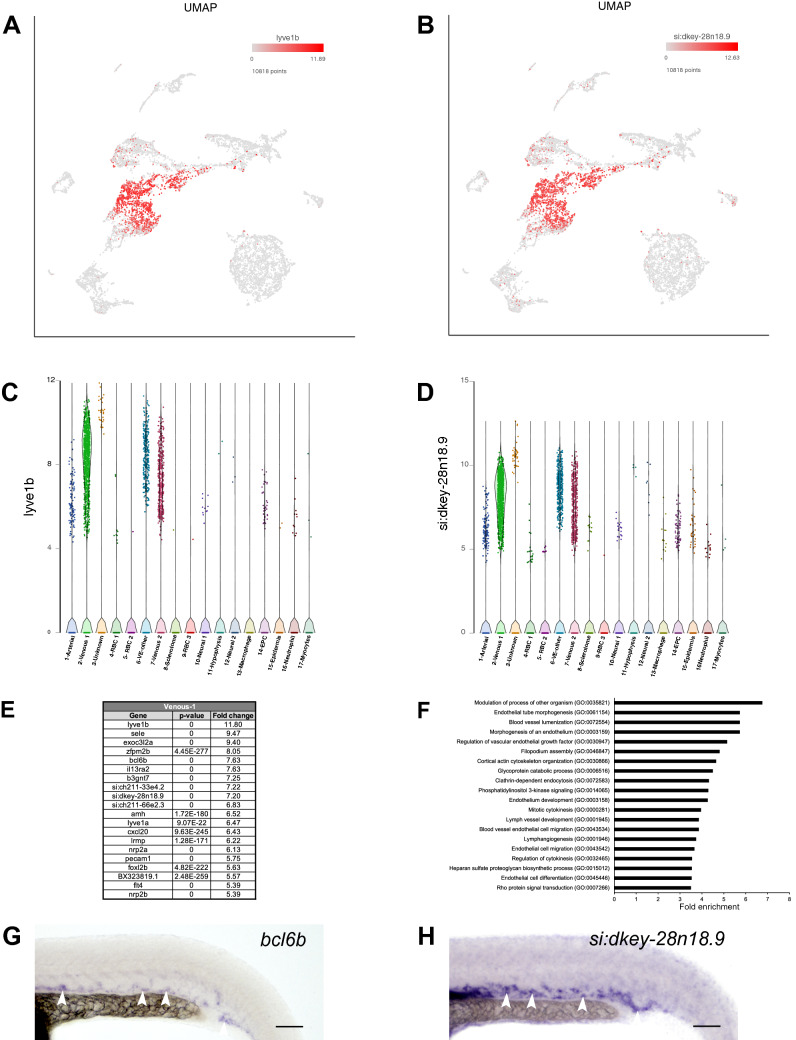
Venous-1 cluster #2. (**A**,**B**) UMAP feature plots showing expression of selected top markers for cluster #2, *lyve1b* and *si:dkey-28n18.9*. (**C**,**D**) Violin plots showing expression of *lyve1b* and *si:dkey-28n18.9* in different cell populations. (**E**) List of top 20 marker genes differentially expressed in Venous-1 cluster. (**F**) List of top 20 pathways enriched in Venous-1 cluster. (**G**,**H**) In situ hybridization analysis at 24 hpf for previously uncharacterized genes *bcl6b* and *si:dkey-28n18.9*. Note the expression of *bcl6b* and *si:dkey-28n18.9* in the posterior cardinal vein (arrowheads). Scale bars: 100 μm.

Top marker genes for cluster #2 included *lyve1b* and *sele*, both known to be enriched in venous cells^31,32^ (Fig. 3A,C and Table S1). Many other known venous marker genes, such as *flt4*, *stab1*, *mrc1a* and *stab2,* were also enriched in cluster #2^33–36^ (Fig. 3E and Table S1). In addition to known venous markers, cluster #2 included many genes with unknown or previously uncharacterized expression pattern, such as *si:dkey-28n18.9* (Fig. 3B,D,E and Table S1). We hypothesized that many of these uncharacterized genes would also be expressed in venous cells. We selected two previously uncharacterized genes, *bcl6b* and *si:dkey-28n18.9,* and analyzed their expression by ISH. Consistent with our hypothesis, we observed expression of *bcl6b* and *si:dkey-28n18.9* in the posterior cardinal vein (PCV) (Fig. 3G,H). Top GO biological processes included endothelial tube morphogenesis, blood vessel lumenization and morphogenesis of an endothelium as well as PI3 signaling and lymphangiogenesis (Fig. 3F and Table S3). Highly represented pathways included PI3 kinase, Insulin/IGF, Integrin and Ras signaling pathways (Table S4). PI3 kinase pathway has been previously implicated in arterial-venous specification^30^, while Insulin/IGF pathway has established roles in angiogenesis^37^. Other top pathways such as Integrin signalling have been shown to play a crucial role in vasculogenesis^28^, while Ras signaling pathway has also been implicated to regulate arterial-venous specification in zebrafish^38^. In addition, VEGF, FGF and Notch signaling pathways, which have established roles in vascular development and arteriovenous differentiation, were also among top pathways identified by the gene list in cluster #2^39,40^ (Table S4).

**Figure 4:**
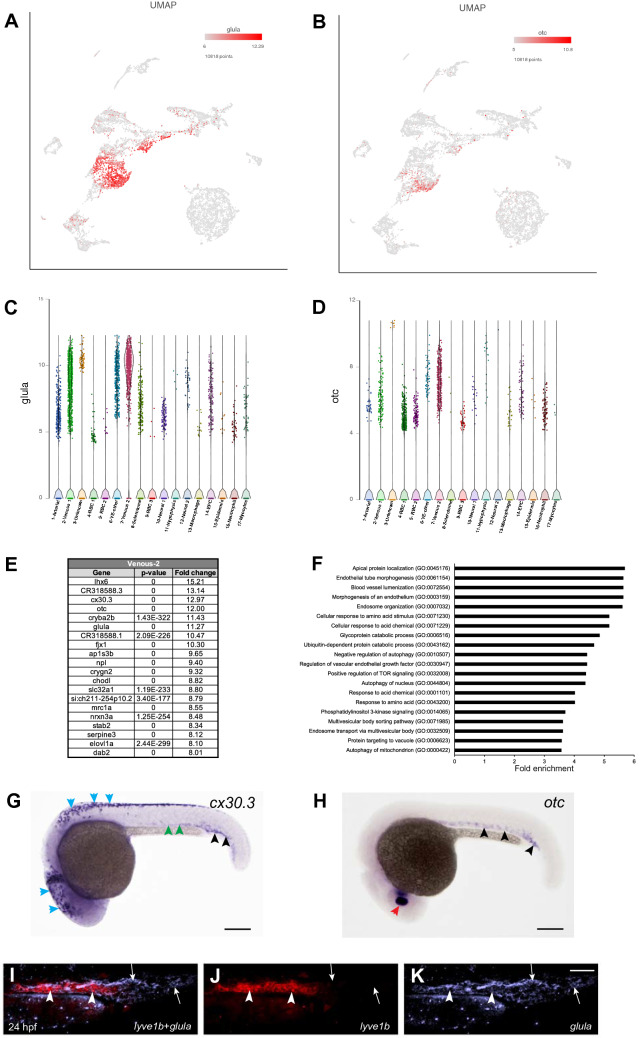
Venous-2 cluster #7. (**A**,**B**) UMAP feature plots showing expression of selected top markers for cluster #7, *glula* and *otc*. (**C**,**D**) Violin plots showing expression of *glula* and *otc* in different cell populations. (**E**) List of top 20 marker genes differentially expressed in Venous-2 cluster. (**F**) List of top 20 pathways enriched in Venous-2 cluster. (**G**) In situ hybridization analysis at 24 hpf for a previously uncharacterized marker gene *cx30.3*. Note the expression of *cx30.3* in the caudal vein (black arrowheads) and weak expression in the PCV (green arrowheads). *cx30.3* is also expressed in melanocytes (blue arrowheads). (**H**) In situ hybridization analysis at 24 hpf for a marker gene *otc*. Note the expression of *otc* in the CV, PCV (black arrowheads) and the lens (red arrowhead). Scale bars: 200 μm. (**I**–**K**) Two color fluorescent ISH analysis for the expression of *lyve1b* and *glula* at the 24 hpf stage. Note that many cells in the CV are positive for *glula* and not *lyve1b* (arrows), while cells with strong *lyve1b* expression in the PCV have only weak *glula* expression (arrowheads). Trunk and tail region is shown, anterior is to the left. *PCV* posterior cardinal vein, *CV* caudal vein. Scale bar in (**K**), 50 μm for (**I**–**K**).

Interestingly, we also identified a second cluster, cluster #7, which showed enriched expression of known venous marker genes. Top marker genes for cluster #7 included *glula*, known to be enriched in venous cells^41^ (Fig. 4A,C,E and Table S1). Other known venous markers, including *mrc1a*, *stab2* and *dab2,* were also enriched in cluster #7^35,36,42^ (Fig. 4E and Table S1). In addition to the known venous markers, top marker genes from this cluster also included many previously uncharacterized/unknown genes, such as *cx30.3* and *otc* (Fig. 4B,D,E and Table S1). We performed ISH to confirm if these genes were expressed in venous cells in zebrafish. Indeed, both *cx30.3* and *otc* demonstrated expression in the PCV and the caudal vein (Fig. 4G,H). *cx30.3* expression was enriched in the caudal vein, and only weak expression in the PCV was apparent. In addition, *cx30.3* showed strong expression in the cranial and trunk neural crest cells, while *otc* showed strong expression in the lens. Highly represented GO biological processes for cluster #7 included terms such as apical protein localization, endothelial tube morphogenesis, blood vessel lumenization, and morphogenesis of an endothelium (Fig. 4F and Table S3). Enriched pathways included Insulin/IGF, Hypoxia-induced factor (HIF), PI3 kinase, and Hedgehog signaling pathways (Table S4), which have been all implicated in several steps of vascular development, including angiogenesis^37,43^, arteriovenous specification^30^, and establishing vascular integrity^44^.

We next asked if there was a significant biological difference between the two separate venous clusters and if they represented two distinct subsets of venous cells. To examine this, we performed fluorescent in situ hybridization for selected top markers of the two venous clusters, *lyve1b* (cluster #2) and *glula* (cluster #7), using *Tg(kdrl:GFP)* embryos at 24 hpf stage. There was a significant overlap between *lyve1b* and *glula* expression within the PCV in the trunk region. Intriguingly, the caudal vein (CV) displayed *glula* expression, while *lyve1b* expression was nearly absent (Fig. 4I–K). In contrast, a subset of endothelial cells within the PCV displayed strong *lyve1b* expression and showed very little *glula* expression (Fig. 4I–K), suggesting heterogeneity in the venous cell population. These results suggest that cluster #7 is enriched in the caudal vein cells. *tfec,* a previously characterized marker for caudal endothelial cells^45^, was preferentially enriched in the cluster #7 (Table S1), supporting its annotation as the caudal vein.

We have previously reported transcriptomic profiles of arterial and venous endothelial cells identified by scRNA-seq analaysis of zebrafish trunks at 30 hpf^46^. A substantial overlap was observed between 24 and 30 hpf datasets in both arterial and venous marker expression (Table S5). While there was only a single venous specific cluster identified at 30 hpf, its top marker genes were shared between 24 hpf Venous-1 and Venous-2 clusters, reflecting a substantial overlap between the two venous clusters and suggesting that many arterial and venous markers identified at 24 hpf continue their specific expression through later stages.

### Identification of vascular endothelial cluster co-expressing both arterial and venous markers

**Figure 5:**
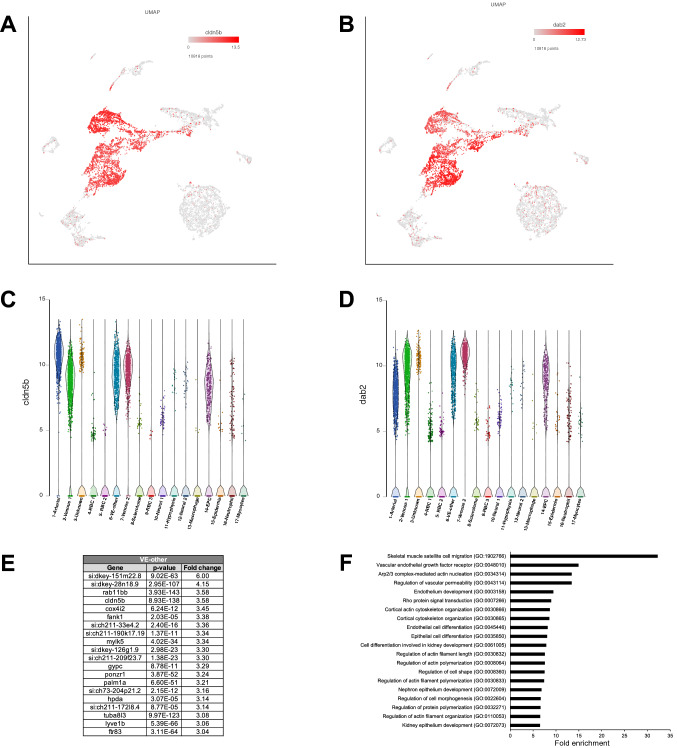
Vascular endothelial (VE)-other cluster #6. (**A**,**B**) UMAP feature plots showing expression of selected markers for cluster #6, *cldn5b* and *dab2*. (**C**,**D**) Violin plots showing expression of *cldn5b* and *dab2* in different cell populations. (**E**) List of top 20 genes differentially expressed in Vascular endothelial (VE)-other cluster. (**F**) List of top 20 pathways enriched in Vascular endothelial (VE)-other cluster.

**Figure 6:**
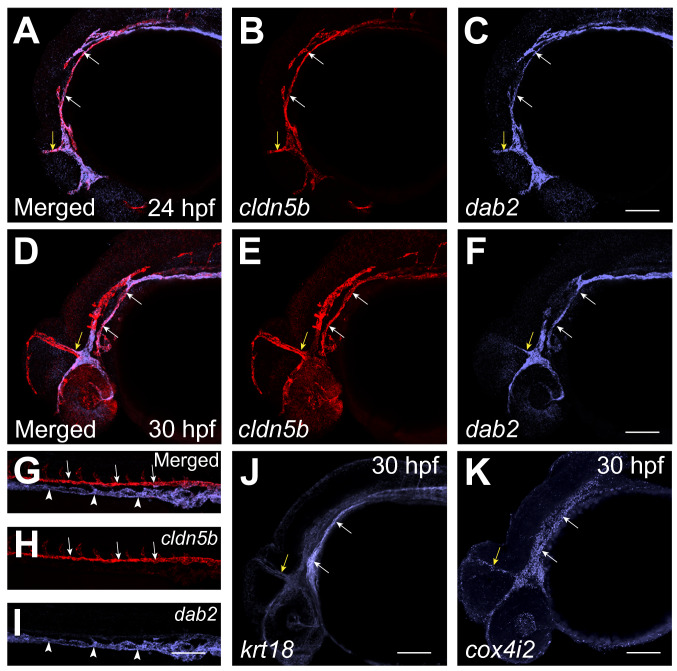
Vascular endothelial (VE)-other cluster #6 corresponds to cells in cranial vasculature. (**A**–**F**) Two color fluorescent ISH analysis for the expression of *cldn5b* and *dab2* at the 24 hpf and 30 hpf stages. Note that *cldn5b* and *dab2* are co-expressed in the PHBC (white arrows) and MCeV (yellow arrows) at the 24 hpf and 30 hpf stages. (**G**–**I**) Two color fluorescent ISH analysis for the expression of arterial *cldn5b* and venous *dab2* in the trunk region of 24 hpf embryos. Note that *cldn5b* and *dab2* have distinct non-overlapping expression in the DA (arrows) and PCV (arrowheads) respectively. (**J**,**K**) Fluorescent ISH analysis for the expression of *krt18* and *cox4i2* in the head region of 30 hpf embryos. Both markers are expressed in the PHBC (white arrows) and MCeV (yellow arrows). *PHBC* primordial hindbrain channels, *MCeV* mid cerebral vein, *DA* dorsal aorta, *PCV* posterior cardinal vein. Scale bars: 100 μm.

Top marker genes for cluster #6 included *cldn5b*, known to be enriched in arterial vascular endothelial cells^47^ (Fig. 5A,C). In addition, known venous markers *dab2* and *lyve1b* as well as pan-endothelial marker *kdrl* were also enriched in cluster #6^31,33^ (Fig. 5B,D,E and Table S1). Other top markers for cluster #6 included *rab11bb, cox4i2, fank1,* and *krt18* (*krt18* had the lowest p value). Expression of these markers either has not been previously characterized or they are known to be expressed in many different cell types, including the vascular endothelium (Table S1). To identify which cells in a zebrafish embryo co-express arterial and venous markers, we performed fluorescent in situ for the arterial marker *cldn5b* and the venous marker *dab2* in *Tg(kdrl:GFP)* embryos. Intriguingly, *cldn5b* and *dab2* showed co-expression within a subset of cranial vasculature including venous primordial hindbrain channels (PHBCs) and mid cerebral vein (MCeV) at 24 and 30 hpf (Fig. 6A–F). In contrast, consistent with previous studies, expression of *cldn5b* and *dab2* was restricted to the DA and PCV, respectively, in the trunk vasculature and did not overlap^48^ (Fig. 6G–I). Other cluster #6 markers *cox4i2* and *krt18* also showed expression in PHBCs and MCeV (Fig. 6J,K). These results suggest that cluster #6 corresponds to a subset of cranial vasculature, which shows a distinct molecular identity from other types of vascular endothelial cells.

Highly represented GO biological processes for cluster #6 included vascular endothelial growth factor receptor, Arp2/3 complex-mediated actin nucleation, regulation of vascular permeability and endothelium development (Fig. 5F and Table S3). Enriched pathways included histamine H2 receptor signaling pathway, implicated in vasodilation in brain capillaries^49^, cytoskeletal regulation by Rho GTPase, implicated in vascular integrity and tubulogenesis^50,51^, and angiotensin II-stimulated signaling through G proteins and beta-arrestin, known to regulate vasoconstriction and blood pressure^52^ (Table S4).

### Identification of endothelial progenitor cells (EPC)

**Figure 7:**
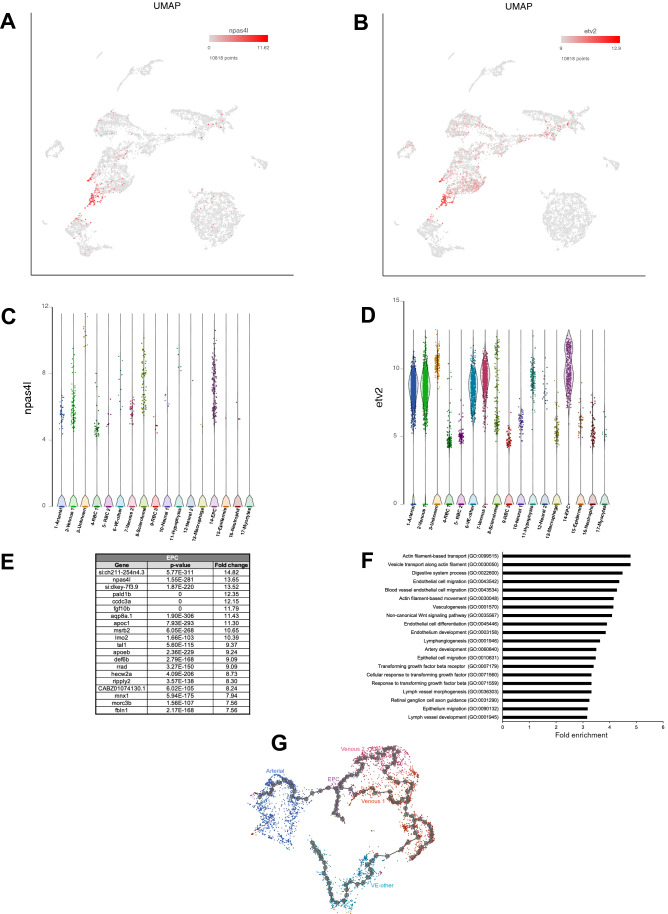
Endothelial progenitor cell (EPC) cluster. (**A**,**B**) UMAP plots showing expression of selected EPC top markers *npas4l* and *etv2*. (**C**,**D**) Violin plots showing expression of selected arterial marker genes *npas4l* and *etv2* in different cell populations. (**E**) List of top 20 genes differentially expressed in the EPC cluster. (**F**) List of top 20 pathways enriched in the EPC cluster. (**G**) Developmental trajectory plot of vascular endothelial cells generated using Monocle 3.

Top marker genes for cluster #14 included *npas4l* and *lmo2*, known to be enriched in endothelial progenitor cells^33,53^ (Fig. 7A,C and Table S1). Other known EPC markers, including *tal1/scl* and *etv2*, were also enriched in cluster #14^15,54^ (Fig. 7B,D,E and Table S1). Highly represented GO biological processes included actin filament-based transport, endothelial cell migration, vasculogenesis, non-canonical Wnt signaling pathway, endothelial cell differentiation, and artery development (Fig. 7F and Table S3). Interestingly, GO biological processes pertaining to lymph vessel development were also enriched. Enriched pathways included PDGF, integrin, TGF-beta and Wnt signaling pathways (Table S4). PDGF signaling pathway has been shown to induce proliferation, migration, and angiogenesis of EPCs^55^. Similarly, Integrin signaling has been shown to be a major regulator of EPC mobilization, homing, invasion and differentiation^56^. TGF-beta and Wnt signaling pathways have been implicated in differentiation and specification of EPCs^57,58^.

To identify developmental relationship between all 5 vascular endothelial clusters, we performed developmental trajectory analysis using Monocle 3^59^ (Fig. 7G). The trajectory analysis suggested that EPC cells transition into 3 distinct branches: an arterial and 2 venous. The two venous branches merged again, perhaps reflecting a significant overlap between the two cell populations. Venous-1 population transitioned into VE-other, which, as our results suggest, corresponds to a subset of cranial vasculature. Some VE-other cells were positioned in the vicinity of arterial population, reflecting mixed arteriovenous identity of VE-other cluster.

### Identification of the endocardial cell cluster

**Figure 8:**
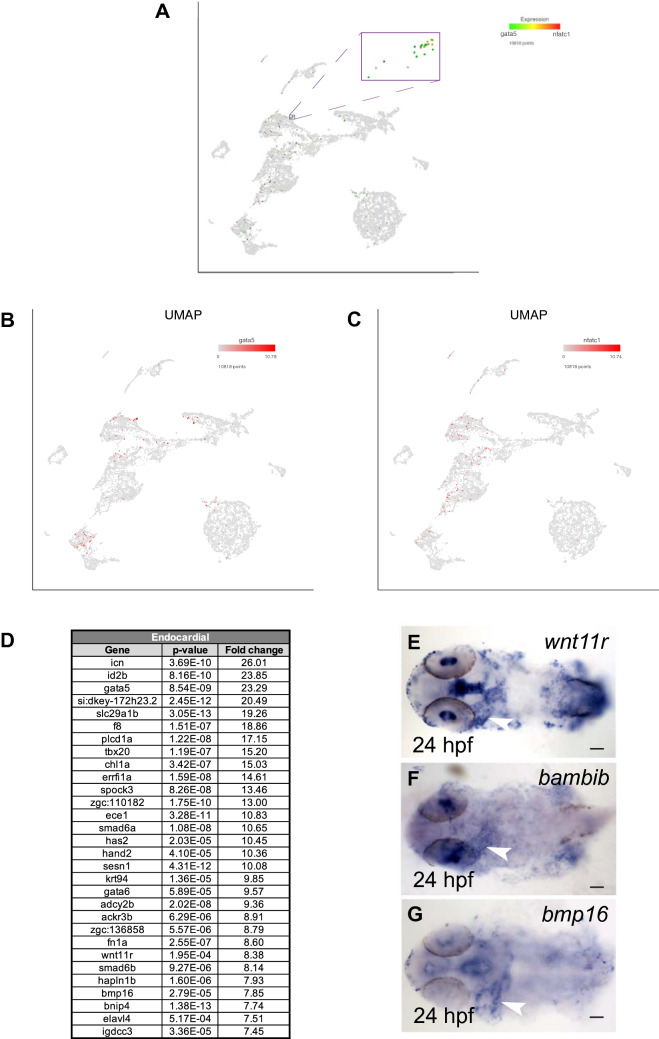
Endocardial subcluster. (**A**) Endocardial cells were identified based on the expression of markers genes *gata5* and *nfatc1* (log_2_ > 0). (**B**,**C**) UMAP feature plots showing expression of *gata5* and *nfatc1*. (**D**) List of top 30 genes differentially expressed in the endocardial subcluster. (**E**–**G**) In situ hybridization analysis at 24 hpf for selected endocardial enriched genes *wnt11r, bambib* and *bmp16*. Note the expression of *wnt11r, bambib* and *bmp16* within the heart tube (arrowhead). Ventral view of deyolked and flat-mounted embryos, anterior is to the left. Scale bars: 100 μm.

While unsupervised clustering identified 5 different endothelial clusters, some other known endothelial cell subtypes, including endocardial cells, lymphatic progenitors, hemogenic endothelium, tip and stalk cells, were not identified using this approach. In an attempt to define additional vascular endothelial cell types, we performed subclustering using vascular endothelial cells only. However, such approach did not identify any additional meaningful clusters beyond what we have already demonstrated. Therefore, we focused on the endocardial cells and attempted to identify their transcriptional profile using the targeted clustering approach based on expression of known marker genes. Endocardial cells are known to share a large transcriptional similarity with other vascular endothelial cells^33,60–63^, but they also have their unique identity known by expression of marker genes such as *nfatc1* and *gata5*^64,65^. Recent studies have described endocardial transcriptome at 15 and 20-somite stages in zebrafish embryos^48,66^. However, endocardial transcriptome at 24 hpf stage has not been previously reported. Therefore, to identify the transcriptomic signature of endocardial cells, we subclustered endothelial cells based on the expression of *nfatc1* and *gata5* (Fig. 8A–C). This resulted in the identification of a single subcluster comprised of 21 cells. Top marker genes for this subpopulation, besides *gata5*, included *has2* and *fn1a*, all known to have endocardial specific expression^64,65,67–69^ (Fig. 8D and Table S6). Known zebrafish endocardial markers *klf2a* and *notch1b* were also enriched in this cluster^70,71^ (Table S6). Heart-specific expression has been reported for many other genes in this cluster including *id2b, tbx20, spock3, ece1* and *smad6a*, although it was not investigated whether this expression was endocardial or myocardial^72–75^. We previously reported the transcriptome of an endocardial cluster identified during scRNA-seq analysis of *etv2*^*ci32Gt*^*; UAS:GFP* embryos at the 20-somite stage (19 hpf)^48^. There was a significant overlap of marker genes, such as *fn1a*, *gata5*, *id2b, wnt11r*, *bmp16* and *bambib* between the two endocardial clusters (Table S7). A recent study reported the zebrafish endocardial transcriptome at the 15-somite stage^66^. There was an extensive overlap between our endocardial dataset and the one reported by Capon et al. (Table S7), further validating endocardial identity of the *nfatc1* + *gata5* + cell cluster. As an additional confirmation, we analyzed expression of 3 selected markers *wnt11r*, *bmp16* and *bambib,* endocardial expression of which has not been previously reported. In situ hybridization confirmed the expression of *wnt11r*, *bambib* and *bmp16* in the heart at 24 hpf (Fig. 8E–G). To gain further insights into the signature of the endocardial cells relative to endothelial cells, we used Monocle 3 to analyze the position of endocardial cells in the trajectory plot. Majority of the endocardial *gata5* + *nfatc1* + cells clustered within or next to the arterial cluster. However, a few endocardial cells were also present within the venous cluster (Figure S2), suggesting a unique signature and heterogeneity between endocardial cells at 24 hpf. Altogether, we have identified the transcriptomic signature of zebrafish endocardial cells and validated new zebrafish endocardium markers.

## Discussion

In this study we used scRNA-seq analysis to identify transcriptomic signatures of vascular endothelial cells in zebrafish embryos at 24 hpf, at the onset of blood circulation. Our profiling of 10,818 cells identified 6 different subtypes of vascular endothelial cells which included vascular progenitor, arterial, two different venous, endocardial, and cranial endothelial subtypes. Importantly, we have validated new markers for arterial, two different venous, and endocardial populations.

Intriguingly, two different venous populations were identified during scRNA-seq analysis. The PCV is known to harbor a heterogenous cell population including progenitors of lymphatic and subintestinal vasculature^76–78^. Many markers including *lyve1b* overlap between venous and lymphatic cells during development^79^ making it difficult to distinguish between the two lineages. *Prox1a* and *prox1b* are considered to be the definitive markers of lymphatic lineage^80–83^. Both markers were similarly enriched in the venous-1 (cluster #2, log_2_ values 2.85 and 3.96, respectively) and venous-2 (cluster #7, log_2_ values 2.97 and 3.46, respectively, Table S1) clusters. Therefore, it is unlikely that either of the clusters corresponds to lymphatic progenitors. Only a small number of cells were positive for either *prox1a* or *prox1b*, and they did not cluster together, preventing us from identifying lymphatic transcriptome at this developmental stage. While subintestinal progenitors also originate from the PCV starting at 28 hpf^76–78^, it is unclear if these cells have a unique transcriptomic signature at these early stages. Our data suggest that the venous-2 population is enriched in markers for the caudal vein. The caudal vein is known to have a unique transcriptional profile^84,85^ and also serves as the niche for hematopoietic stem cells^86^. Therefore, transcriptional characterization of caudal vein endothelium will help to understand pathways involved in its development.

An identity of cluster #6 (VE-other) is the most nebulous. Many top marker genes for this cluster are poorly characterized. Marker genes with known endothelial specific expression pattern include pan-endothelial *kdrl*, arterial *cldn5b* and venous specific *lyve1b* and *dab2* (Table S1). Although the spatial and temporal origin of arteries and veins has been well established, the precise timing when arterial and venous-fated precursor cells differentiate into distinct arterial and venous vessels has been controversial^87–89^. We have previously shown co-expression of venous and arterial genes in many arterial progenitors at the 20-somite stage in the trunk region of zebrafish embryos. However, at later stages of development (24 hpf) the expression of venous and arterial markers becomes restricted to veins and arteries, respectively^48^, in the trunk vasculature. In contrast, we show here that arterial and venous genes are co-expressed in a subset of cranial vasculature including PHBC and MCeV at 24 hpf as well as 30 hpf. These results suggest mixed arteriovenous identity of cranial vasculature during embryonic development. Cells within cranial vasculature also show strong expression of other cluster #6 markers, such as *cox4i2* and *krt18*, further supporting the argument that cluster #6 corresponds to a subset of cranial vasculature. However, we cannot rule out the possibility that cells with similar gene expression signature are also located elsewhere in the embryo. It is also possible that such expression pattern may represent a transitory phase of differentiation during embryonic development.

Endocardial cells share many similarities with vascular endothelial cells. However, they are also known to harbor a unique transcriptomic signature as evident by the expression of *nfatc1*^64,90^. Differences in the transcriptomes between endocardial and other types of vascular endothelial cells have not been well characterized during embryonic development, and only a limited number of endocardial-specific markers are currently known in any vertebrate model system. We have previously reported a transcriptional signature of zebrafish endocardial progenitors identified during scRNA-seq analysis of *etv2* reporter embryos at the 20-somite stage (19 hpf)^48^. In addition, a recent study performed RNA-seq using an endocardial specific reporter line to describe the endocardial transcriptome at the 15-somite stage^66^. Here we performed subclustering based on the established markers *gata5* and *nfatc1* to define the endocardial transcriptome at 24 hpf. A significant overlap between the endocardial marker genes at 15-somite, 20-somite and 24 hpf stages was observed which confirms the endocardial identity of *gata5* + *nfatc1* + cells. Multiple genes in this subcluster have been previously reported to show heart-specific expression, although most studies did not distinguish between the endocardial and myocardial expression. We further validated and demonstrated cardiac expression for 3 previously uncharacterized genes from this subcluster. Identification of the endocardial signature will be a useful resource for many investigators involved in cardiovascular research.

In summary, we identified transcriptional signatures of vascular endothelial cells during zebrafish development and described six different subtypes of endothelial-related cells at 24 hpf, when blood circulation is first initiated. This is one of the first reported scRNA-seq datasets of vascular endothelial cells during zebrafish development. We expect that this will be a useful resource for many researchers in cardiovascular development and will promote investigation of molecular mechanisms involved in the establishment of vascular heterogeneity.

## Materials and methods

### Zebrafish lines and maintenance

All studies were performed following standard guidelines and protocols approved by the University of South Florida and Cincinnati Children’s Hospital Medical Center Institutional Animal Care and Use Committee. Embryos were raised at 28.5 °C in embryo medium and staged by hours post fertilization (hpf)^91^. Zebrafish lines used in the study are as follows: wild-type AB, wild-type EKK, *Tg(kdrl:GFP)*^*s843*^^92^, *Tg(kdrl:mCherry)*^14^ and *Tg(etv2*^*ci32Gt*^*; UAS:GFP*)^18^.

### Embryo dissociation and single-cell RNA-seq

*Tg(kdrl:mCherry)* fish were crossed to *etv2*^*ci32Gt*^*; UAS:GFP* line. Embryos double positive for both mCherry and GFP fluorescence were selected at 24 hpf. Embryos were dechorionated and immediately transferred to a 1.5 ml Eppendorf tube placed on ice for the generation of single-cell suspension. Whole embryos were then dissociated into a single-cell suspension by cold protease tissue dissociation protocol^93^. Cells expressing GFP only and both GFP and mCherry were sorted using a BD/FACSAria II flow cytometer cell sorter. A suspension of ~ 10,000 single cells was loaded onto the 10XGenomics Single Cell 3’ (V3 chemistry) chip at the CCHMC Gene Expression Core. 12 cDNA amplification cycles were used to generate cDNA. Samples were sequenced on an Illumina NovaSeq 6000 instrument (Illumina, San Diego, CA) running an S2 flow cell with parameters as follows: Read1, 28 cycles; Index Read1: 8 cycles; Read 2: 91 cycles at the CCHMC DNA Sequencing core. The raw Fastq files obtained from the sequencing core were then mapped to the *Danio rerio* genome (version zv11) to generate single-cell feature counts using Cell Ranger version 3.0.2. Counts were performed on fastq data from each of the populations individually. Filtered matrix files were then imported into Partek Flow analysis Suite. Cells were filtered on total read per cell (477–61,000), expressed genes per cell (96–5456) and mitochondrial counts (0–10%) which resulted in 10,818 cells. The gene count CPM (counts per million) values were normalized by adding 1.0 and calculating log_2_ values. GFP + and GFP + mCherry + samples were combined, and an unbiased graph-based clustering was performed. Finally, UMAP plots, violin plots, scatter plots, a dot plot, and a heatmap were obtained and exported from Partek. Endocardial cluster was identified based on the expression of marker genes *gata5* and *nfatc1* (log_2_ *gata5* and *nfatc1* > 0).

#### Gene ontology (GO) and pathway analysis

GO analysis was performed by applying gene list from respective clusters into GO site http://geneontology.org/ (GO Ontology database https://doi.org/10.5281/zenodo.6399963 Released 2022–03-22). To perform pathway analysis, PANTHER overrepresentation test (PANTHER version 17.0 Released 2022–02-22) was used. Statistical significance was calculated by Fisher’s exact test. A False discovery rate (FDR) corrected P-values less than 0.05 were considered statistically significant.

#### WISH and HCR

Whole mount in situ hybridization was performed as described before^94^.

To synthesize *bcl6b, cx30.3, esm1, otc* and *si:dkey-28n18.9* probes, partial cDNA was first amplified from the cDNA of 24 hpf embryos using reverse oligo containing T7 promoter sequence. Resulting PCR product was then used as a template for RNA synthesis. Following primers were used for amplification of partial cDNA (Table S8). Anti-sense RNA was synthesized using T7 RNA polymerase (Promega) and DIG-labeling mix (Sigma-Aldrich). To synthesize *bambib, bmp16, notchl* and *wnt11r* probes, partial cDNA sequence was amplified using gene specific primers (Table S8). cDNA for each gene was subcloned into PCRII-TOPO vector using TOPO T/A cloning (ThermoFisher). Plasmids were linearized and antisense RNA was synthesized using SP6 *(bmp16, wnt11r)* or T7 *(bambib, notchl)* RNA polymerase (Promega) and DIG-labeling mix (Sigma-Aldrich). WISH embryos were imaged with a Nikon Eclipse Ni-E or Zeiss Axio imager compound microscope. HCR v3.0 probes for *cldn5b*, *dab2*, *lyve1b*, *glula* and *krt18* were obtained from Molecular Technologies, Inc., Los Angeles, CA, USA. HCR in situ was performed as described previously^95^. Embryos were mounted in 0.6% low-melting agarose and imaged using Nikon Eclipse confocal microscope. At least 15 embryos processed for WISH and HCR were analyzed.

### Ethical approval

The study is reported in accordance with ARRIVE guidelines^96^.

## Supplementary Information

Supplementary Figures.

Supplementary Table S1.

Supplementary Table S2.

Supplementary Table S3.

Supplementary Table S4.

Supplementary Table S5.

Supplementary Table S6.

Supplementary Table S7.

Supplementary Table S8.

## Data Availability

The scRNA-seq datasets generated and/or analyzed during the current study are available in the NCBI GEO database under accession number GSE202912. All processed image datasets are included in this published article (and its Supplementary information files). Original image datasets generated during this study are available from the Lead contact upon reasonable request.
