# Supplementary material for: Single-cell transcriptomic analysis of vascular endothelial cells in zebrafish embryos
Source: Sci Rep. 2022 Jul 29;12:13065. doi: 10.1038/s41598-022-17127-w (PMC9338088; doi:10.1038/s41598-022-17127-w)
Supplement: Supplementary file 1 — Supplementary Figures. [file 41598_2022_17127_MOESM1_ESM.pdf]

Supplementary Data for Gurung et al.

Title of the manuscript: Single-cell transcriptomic analysis of vascular endothelial cells in zebrafish embryos

Authors: Suman Gurung, Nicole K. Restrepo, Brendan Chestnut, Laurita Klimkaite, and Saulius Sumanas

Figures S1 and S2.

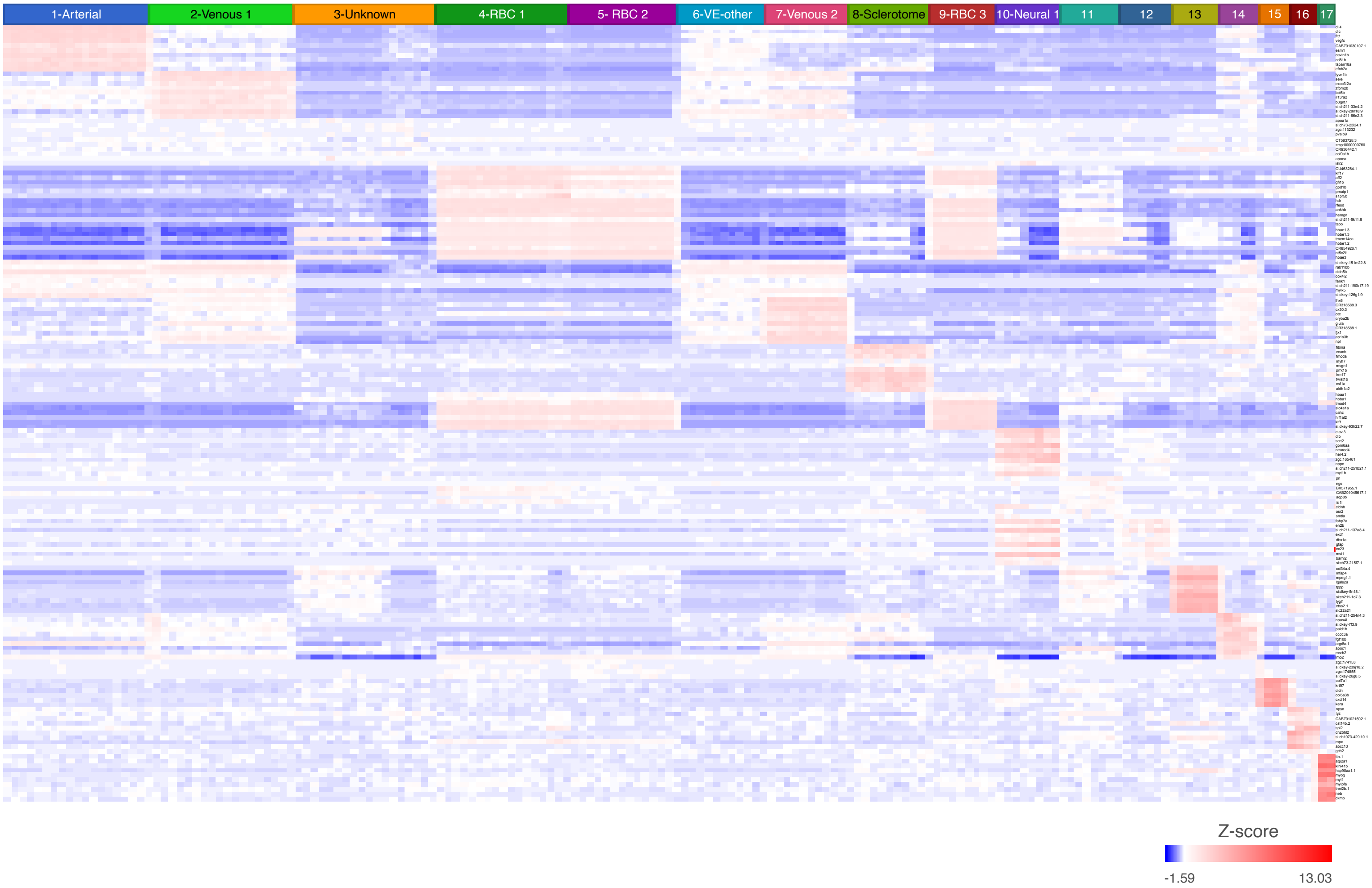

Figure S1. A heatmap showing expression of top marker genes in different clusters. The heatmap was generated using the top 10 marker genes for each clusters. This is an enlarged image from Fig. 1D.

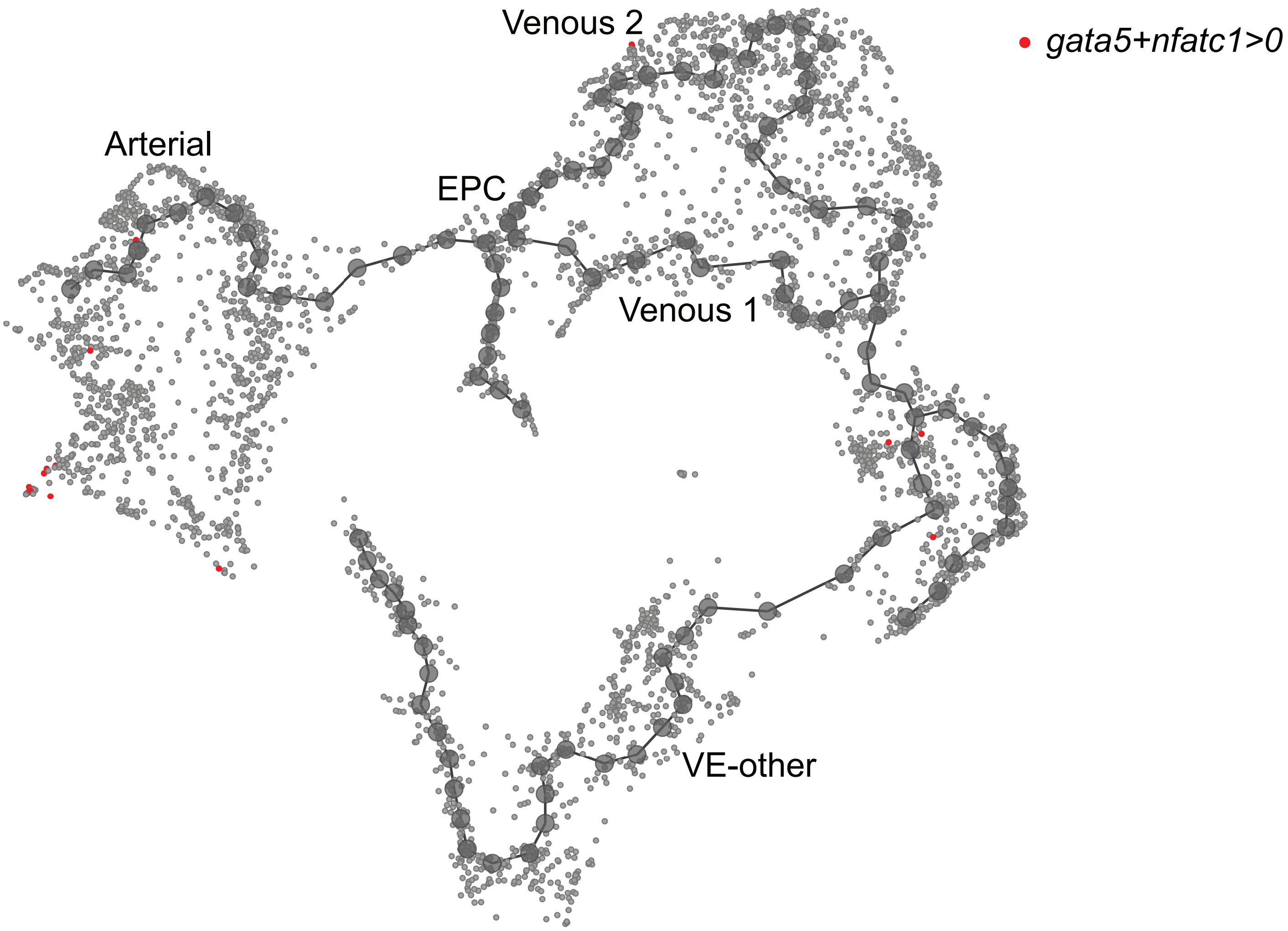

Figure S2. Location of endocardial cells (marked in red), identified based on *gata5* and *nfatc1* expression, within the developmental trajectory plot. Note that majority of the endocardial cells are present next to or within the arterial cluster.
